# MRI findings of epithelial–myoepithelial carcinoma of the parotid gland with radiologic–pathologic correlation

**DOI:** 10.1007/s11604-021-01243-0

**Published:** 2022-01-04

**Authors:** Taketo Suto, Hiroki Kato, Masaya Kawaguchi, Kazuhiro Kobayashi, Tatsuhiko Miyazaki, Tomohiro Ando, Yoshifumi Noda, Fuminori Hyodo, Masayuki Matsuo, Hiromasa Ishihara, Takenori Ogawa

**Affiliations:** 1 Department of Radiology, Gifu University, 1-1 Yanagido, Gifu, 501-1194 Japan; 2 Department of Pathology, Gifu University, Gifu, Japan; 3 Department of Otolaryngology, Gifu University, Gifu, Japan

**Keywords:** Epithelial–myoepithelial carcinoma, Parotid gland, MRI

## Abstract

**Purpose:**

This study aimed to describe the MRI findings of epithelial-myoepithelial carcinoma (EMC) of the parotid gland.

**Materials and methods:**

Seven patients (four males and three females) aged 40–86 years (mean age, 64 years) with histologically proven EMC of the parotid gland who underwent surgical resection after preoperative MRI were enrolled. MRI images were retrospectively reviewed and contrasted with pathological findings.

**Results:**

Five patients (71%) had predominantly solid lesions, and two (29%) had predominantly cystic lesions. All seven lesions had well-demarcated margins and capsules without the invasion of adjacent structures. The capsules were incomplete in five lesions (71%) and complete in two (29%). Four lesions (57%) exhibited a multinodular structure with internal septa. Cystic components were observed in three lesions (43%). On T1-weighted images, the solid components were frequently homogeneous (5/7, 71%), and demonstrated isointensity in five lesions (71%) and hypointensity in two (29%) relative to the spinal cord. On T2-weighted images, the solid components were usually heterogeneous (6/7, 86%), and demonstrated hyperintensity in five lesions (71%) and isointensity in two (29%) relative to the spinal cord. The mean apparent diffusion coefficient value of the solid components was 0.967 × 10^−3^ mm^2^/s.

**Conclusion:**

Parotid gland EMCs usually appeared as predominantly solid lesions with well-demarcated margins and capsules. A multinodular structure with internal septa was characteristics of EMCs.

## Introduction

Epithelial–myoepithelial carcinoma (EMC) is a rare subtype of malignant salivary gland tumor that is composed of a biphasic arrangement of inner luminal ductal cells and outer myoepithelial cells. In 1991, the World Health Organization classification recognized EMC as a distinct entity and subtype of salivary gland adenocarcinoma. The incidence of EMC accounts for approximately 1% of salivary gland tumors. It predominantly occurs in females with a female-to-male ratio of 1.5:1. The average age of onset is approximately 60 years and > 70% of patients with EMC are > 50 years old. The parotid gland is the most common site of EMC; however, it might be encountered in the submandibular and minor salivary glands, such as those in the palate, maxillary sinus, oropharynx, and tongue base [1–3]. The tumors are from 0.5 to 20 cm in size. EMC is usually regarded as a low-grade malignancy with a history of slow growth. It typically presents as a painless mass and rarely presents with facial nerve palsy. Lymph node and distant metastasis rates are low; however, local recurrence is not uncommon [3]. Currently, histopathological examination is required for the diagnosis of EMC and the role of imaging is limited to the evaluation of tumor localization, extension, and metastasis. Although CT and MRI findings of EMCs of the parotid gland have been described in several case reports [4–7], to the best of the authors’ knowledge, no previous studies have summarized the MRI features of EMC. Therefore, this study was performed to determine the MRI characteristics of EMC of the parotid gland with radiologic–pathologic correlation.

## Methods

### Patients

The present study was approved by the human research committee of the institutional review board of our hospital and complied with the guidelines of the Health Insurance Portability and Accountability Act of 1996 and the Declaration of Helsinki. Due to the retrospective nature of the study, the requirement for informed consent was waived. Our hospital’s electronic medical record system was searched for patients with histologically proven EMC of the parotid gland who underwent surgical resection after preoperative MRI between March 2008 and June 2020. In total, seven patients with histologically proven EMC of the parotid gland were enrolled in this study (four males and three females) with an age range of 40–86 years (mean age, 64 years). The seven patients initially presented with a painless mass of the parotid gland without facial nerve palsy. Based on a preoperative assessment, lymph node and distant metastasis were not confirmed. However, local recurrence and cervical lymph node metastasis occurred in one patient during follow-up after surgical resection.

### Imaging technique

MRI was performed using a 3.0-T MRI system (Intera Achieva 3.0 T Quasar Dual; Philips Healthcare, Best, The Netherlands) in five patients or a 1.5-T MRI system (Intera Achieva 1.5 T Pulsar; Philips Healthcare, Best, The Netherlands) in the remaining two patients. All transverse MRI images were obtained at a section thickness of 3–4 mm with 1 mm intersection gap. T2-weighted fast spin-echo images (TR/TE, 3210–5710/90 ms; field of view, 20 × 20 cm), T1-weighted spin-echo images (TR/TE, 687–827/9–18 ms; field of view, 20 × 20 cm), and diffusion-weighted (DW) short-tau inversion recovery (STIR) single-shot spin-echo echo-planar (TR/TE/TI, 5490–18,580/70–72/170–240 ms; field of view, 24 × 24–40 × 40 cm; *b* value, 0 and 1000 s/mm^2^) images were obtained in all patients. Gadolinium-enhanced fat-suppressed T1-weighted spin-echo images (TR/TE, 630–652/9–15 ms; field of view, 20 × 20 cm) were obtained after the intravenous injection of 0.1 mmol/kg of gadopentetate dimeglumine (Magnevist; Bayer Healthcare, Leverkusen, Germany) or gadobutrol (Gadavist, Bayer HealthCare, Leverkusen, Germany) in five patients.

### Imaging assessment

Two radiologists with 22 and 8 years of post-training experience in head and neck imaging reviewed all of the MRI images while unaware of the clinical information. Any disagreement between the radiologists was resolved by reaching a consensus through discussion.

For qualitative assessment, the reviewers assessed the location. The parotid gland was divided into superficial and deep lobes based on the retromandibular vein. The superior and inferior poles were defined as the superior and inferior half of the parotid gland, respectively. The tumor margins were classified as either well- or ill-demarcated. The presence of capsules, which were defined as a hypointense rim on T2-weighted images, was also assessed. In addition, the qualitative internal characterizations of MRI images for the presence of multinodular structure, internal septa, and cystic components were also evaluated. On T2-weighted images, the internal septa were defined as hypointense linear structures within the tumors. The cystic components were defined as unenhanced areas on contrast-enhanced T1-weighted images. If contrast-enhanced imaging was not performed, the cystic components were defined as T2 hyperintense areas with high apparent diffusion coefficient (ADC) values and/or hyperintense areas on T1-weighted images. The reviewers also assessed the predominance of cystic or solid components. The heterogeneities of the solid components were assessed using T1-, T2-, and contrast-enhanced fat-suppressed T1-weighted images and classified as either homogeneous or heterogeneous. The signal intensities of the solid and cystic components relative to the spinal cord on T1- and T2-weighted images were qualitatively assessed. The degrees of contrast enhancement were qualitatively classified as mild, moderate, or marked. These internal signal intensities of the solid components, except the internal septa, were assessed.

Then, quantitative data from the MRI images for the maximum diameter of the tumors and ADC values of the solid components were measured. ADC values (× 10^−3^ mm^2^/s) were measured on ADC maps by placing ROIs over the tumors. ROIs on ADC maps were placed within the solid components as broadly as possible, while excluding the cystic components by referring to T2- and/or gadolinium-enhanced T1-weighted images.

### Histopathological assessment

A pathologist with 12 years of post-training experience macroscopically and microscopically reviewed the surgically resected specimens of the patients with EMC of the parotid gland. The pathologist carefully classified the margin as either well- or ill-demarcated and assessed the presence of fibrous capsule, multinodular structure, internal septa, cystic degeneration, necrosis, hemorrhage, myxoid matrix, and chondroid matrix. The presence of myxoid matrix was evaluated by periodic acid-Schiff staining, whereas that of chondroid matrix was evaluated by Alcian blue staining.

## Results

### MRI findings

**Fig. 1:**
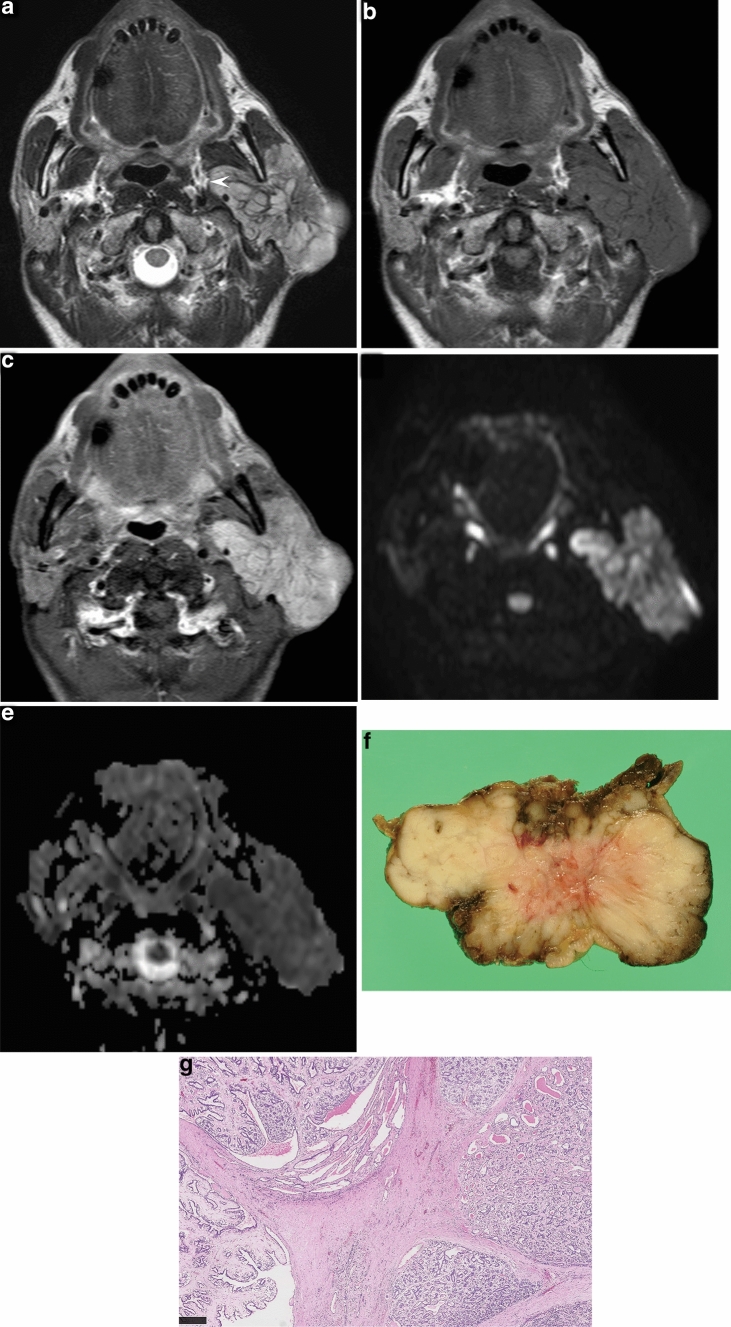
A 40-year-old man with epithelial-myoepithelial carcinoma of the left parotid gland (case 1). **A** T2-weighted image (TR/TE, 5710/90 ms) shows a predominantly solid, well-demarcated lesion with a partial capsule (arrowhead). Multinodular structure with internal septa was clearly demonstrated. The tumor shows heterogeneous hyperintensity relative to the spinal cord. **b** T1-weighted image (TR/TE, 779/15 ms) shows a homogeneously isointense lesion relative to the spinal cord. **c** Contrast-enhanced fat-suppressed T1-weighted image (TR/TE, 632/15 ms) shows a homogeneously, moderately enhanced lesion. **d** Diffusion-weighted image (TR/TE/TI, 18,580/70/240 ms) shows a mildly hyperintense lesion. **e** ADC map shows relatively low ADC value (1.137 × 10^−3^ mm^2^/s). **f** On gross examination, the cut surface shows a solid and lobulated mass, which looks light tan in color, accompanied by multinodular structure with internal septa. The multinodular structure and internal septa are accurately reflected in MRI. **g** Microscopic histological specimen (H&E stain, × 40) shows a multinodular structure with intervening fibrous septa

**Fig. 2:**
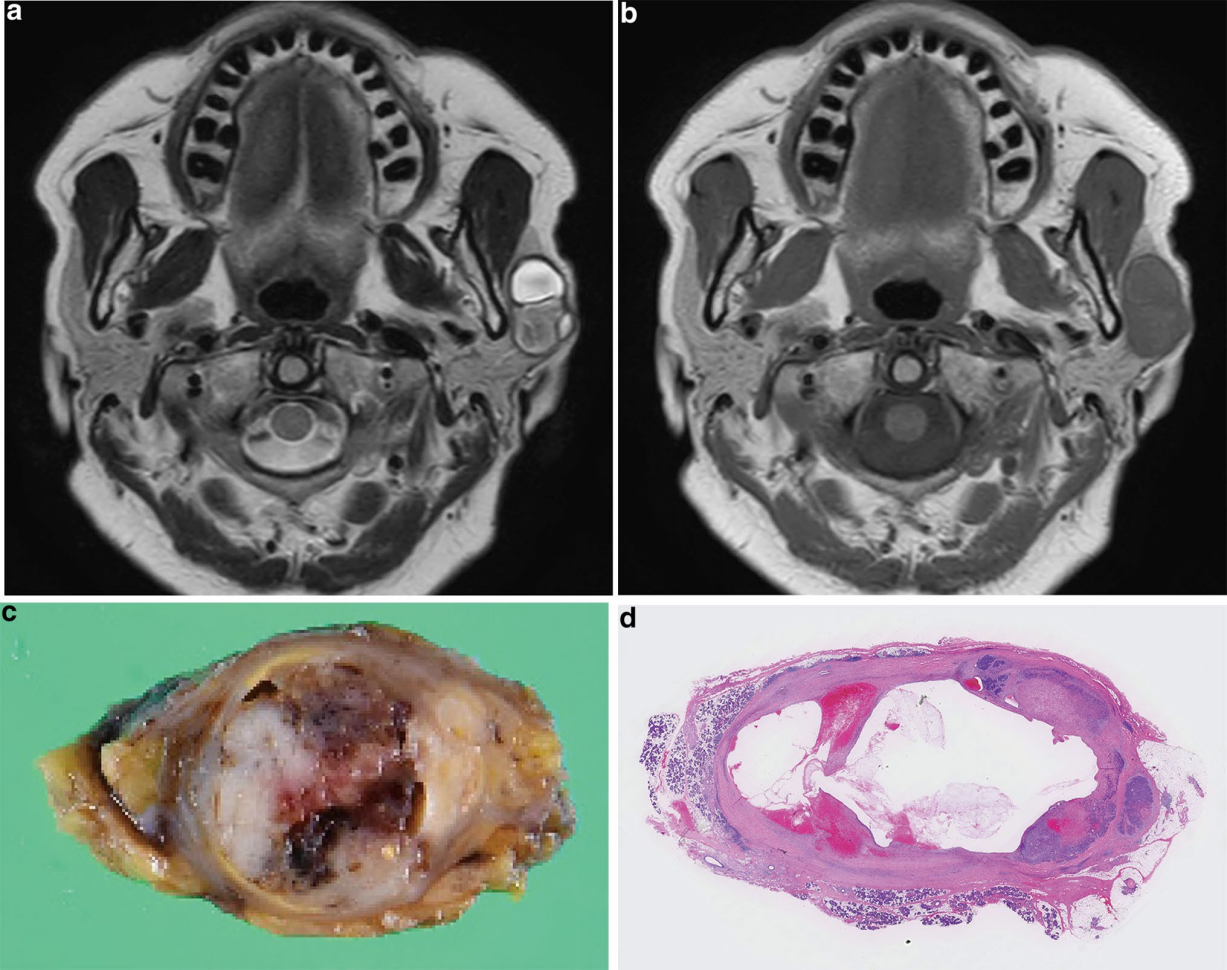
A 68-year-old woman with epithelial-myoepithelial carcinoma of the left parotid gland (case 4). **a** T2-weighted image (TR/TE, 3680/90 ms) shows a predominantly cystic, well-demarcated, multiloculated lesion with a complete capsule. **b** T1-weighted image (TR/TE, 686/18 ms) shows a homogeneously, slightly hyperintense lesion relative to the spinal cord. **c** On gross examination, the cut surface showed a cystic mass, which looks grayish white in color, accompanied by solid components. **d** Macroscopic histological specimen (H&E stain) shows a cystic mass with mural nodule, hemorrhage, and thick fibrous capsule. The cystic appearance and fibrous capsule are accurately reflected in MRI

The MRI characteristics of EMCs are summarized in Tables 1 and 2. The maximum diameter was 10–70 mm (mean, 29.1 mm). The common locations of the tumors were the superficial lobe (6/7, 86%) and superior pole (5/7, 71%). All seven lesions had well-demarcated margins and capsules without the invasion of adjacent structures. The capsules were incomplete in five lesions (71%) and complete in two (29%). Four lesions (57%) exhibited a multinodular structure with internal septa. Cystic components were observed in three lesions (43%). Five patients (71%) had predominantly solid lesions (Fig. 1), and the remaining two (29%) had predominantly cystic lesions (Fig. 2).

**Table 1:** MR findings of seven patients with epithelial-myoepithelial carcinoma of the parotid gland

Case no	Age (year)/sex	MD (mm)	Margin	Capsule	Multinodular structure	Septa	Predominance	Solid components on T1-weighte images		Solid components on T2-weighte images		ADC value (× 10^−3^ mm^2^/s) of solid components	Contrast enhancement of solid components
								Homogeneity	Signal^a^	Homogeneity	Signal^a^		
1	40/M	70	Well	Partial	+	+	Solid	Homo	Iso	Hetero	High	1.137	Heterogenous/moderate
2	45/F	10	Well	Partial	−	−	Solid	Homo	Low	Homo	Iso	0.957	NA
3	66/M	25	Well	Complete	+	+	Cystic	Hetero	Iso	Hetero	High	0.789	Heterogenous/moderate
4	68/F	27	Well	Complete	+	+	Cystic	Homo	Iso	Hetero	High	0.899	NA
5	70/M	16	Well	Partial	−	−	Solid	Homo	Iso	Hetero	High	0.965	Homogenous/mild
6	73/M	23	Well	Partial	−	−	Solid	Homo	Iso	Hetero	Iso	0.939	Heterogenous/moderate
7	86/F	33	Well	Partial	+	+	Solid	Hetero	Low	Hetero	High	1.081	Heterogenous/moderate

The solid components were assessed for homogeneity, signal intensity, and ADC value

*M* male, *F* female, *MD* maximum diameter, *well* well-demarcated, *septa* internal septa, *homo* homogeneous, *hetero* heterogeneous, *signal* signal intensity, *iso* isointensity, *low* hypointensity, *high* hyperintensity, *ADC* apparent diffusion coefficient, *NA* not available

^a^Signal intensities on T1- and T2-weighted images were compared with the spinal cord

**Table 2:** Summary of MR features of epithelial-myoepithelial carcinoma of the parotid gland

*MR imaging characteristics*	
Maximum diameter (mm)	
Mean (range)	29.1 (10–70)
Location	
Left:right	6:1
Superficial:deep:superficial to deep lobe	6:0:1
Superior:inferior:superior to inferior pole	5:1:1
Well-demarcated margins	7 (100%)
Capsule	7 (100%)
Complete:partial	2: 5
Multinodular structure with internal septa	4 (57%)
Cystic components	3 (43%)
Predominance	
Solid:cystic	5:2
Solid components	
T1-weighted images	
Homogenous:heterogenous	5:2
Hypo-:iso-:hyperintensity^a^	2:5:0
T2-weighted images	
Homogenous:heterogenous	1:6
Hypo-:iso-:hyperintensity^a^	0:2:5
Contrast-enhanced images (*n* = 5)	
Homogenous:heterogenous	1:4
Mild:moderate:marked enhancement	1: 4: 0
Cystic components (*n* = 3)	
T1-weighted images	
Hypo-:iso-:hyperintensity^a^	0:1:2
T2-weighted images	
Hypo- to hyper-:iso- to hyper-:hyperintensity^a^	1:1:1
ADC value of solid components (× 10^−3^ mm^2^/s)	
Mean (range)	0.967 (0.789–1.137)

Quantitative variables are expressed as means; numbers in parentheses are ranges. Qualitative variables are expressed as raw numbers; numbers in parentheses are proportions followed by percentages

*ADC* apparent diffusion coefficient

^a^Signal intensities on T1- and T2-weighted images were compared with the spinal cord

On T1-weighted images, the solid components were frequently homogeneous (5/7, 71%), and demonstrated isointensity in five lesions (71%) and hypointensity in two (29%) relative to the spinal cord. On T2-weighted images, the solid components were usually heterogeneous (6/7, 86%), and demonstrated hyperintensity in five lesions (71%) and isointensity in two (29%) relative to the spinal cord. In five lesions that underwent contrast-enhanced imaging, the solid components on contrast-enhanced fat-suppressed T1-weighted images were usually heterogeneous (4/5, 80%), and demonstrated moderate enhancement in four lesions (80%) and mild enhancement in one (20%).

In three lesions with cystic components, the cystic components on T1-weighted images demonstrated hyperintensity (2/3, 67%) and isointensity (1/3, 33%) relative to the spinal cord. On T2-weighted images, the cystic components demonstrated hypo- to hyperintensity (1/3, 33%), iso- to hyperintensity (1/3, 33%), and hyperintensity (1/3, 33%) relative to the spinal cord.

The ADC value of the solid components was from 0.789 to  1.137 × 10^−3^ mm^2^/s (mean, 0.967 × 10^−3^ mm^2^/s).

### Histopathological findings

The histopathological characteristics of EMCs are summarized in Table 3. Five lesions had well-demarcated margins (71%), while the remaining two had ill-demarcated margins (29%). Fibrous capsules, multinodular structures, and internal septa were observed in six lesions (86%). The histological specimens revealed cystic degeneration in two lesions (29%), necrosis in one (14%), and hemorrhage in four (57%). All seven lesions had myxoid and chondroid matrices.

**Table 3:** Histopathological findings of seven patients with epithelial-myoepithelial carcinoma of the parotid gland

Case no	Age (year)/sex	Margin	Capsule	Multinodular structure	Septa	Cystic degeneration	Necrosis	Hemorrhage	Myxoid matrix	Chondroid matrix
1	40/M	Well	+	+	+	−	−	−	+	+
2	45/F	Well	+	+	+	−	−	−	+	+
3	66/M	Well	+	+	+	+	−	+	+	+
4	68/F	Well	+	+	+	+	+	+	+	+
5	70/M	Ill	−	+	−	−	−	−	+	+
6	73/M	Well	+	−	+	−	−	+	+	+
7	86/F	Ill	+	+	+	−	−	+	+	+

*M* male, *F* female, *MD* maximum diameter, *well* well-demarcated, *ill* ill-demarcated

### Radiologic–pathologic correlation

T2-weighted images could demonstrate hypointense rims in all lesions, whereas histological investigation revealed fibrous capsules in six lesions and surrounding fibrous tissues in the remaining one. The radiological margins were well-demarcated in all lesions, whereas the histological margins were ill-demarcated in two lesions because of microscopic extension beyond the capsule or fibrous tissue. The frequency of multinodular structures (86% vs. 57%) and internal septa (86% vs. 57%) was higher on histological assessment than on MRI due to different spatial resolutions. In one lesion (case 7), although cystic degeneration and necrosis were not observed on histological specimens, the reviewers observed marked hyperintensity on T2-weighted images caused by abundant chondroid matrix as cystic components.

## Discussion

EMCs should be diagnosed based on conventional optical microscopy and immunohistochemistry. Based on the histopathological assessments, EMCs were composed of small ducts, and some of which contained small quantities of intraluminal inspissated mucin. EMCs were characterized by biphasic tubular structures composed of inner small cuboidal or low columnar cells with generally eosinophilic cytoplasm and were surrounded by an outer layer of cells that mostly had a clear cytoplasm. The histological differential diagnosis of EMCs includes all other salivary gland neoplasms, such as pleomorphic adenoma, myoepithelial carcinoma, adenoid cystic carcinoma, acinic cell carcinoma, mucoepidermoid carcinoma, and metastatic renal cell carcinoma [8].

Several previous case reports described the CT and MRI findings of EMCs of the parotid gland [4–7]. Based on these case reports, EMCs appear as either predominantly solid lesions [4, 5, 7] or as predominantly cystic lesions [6]. The interface between the tumor and parotid parenchyma was clearly defined in all cases [4–7]. A multinodular appearance with internal septa on T2-weighted images was observed in more than half of previous cases [4, 5]. The frequency of these morphological characteristics was almost identical to the results of the present study. In this study, the signal intensities of the solid components on T1-weighted images were frequently homogeneous, whereas they were often heterogeneous in previously reported cases, especially in larger tumors [4, 5, 7]. The common signal intensities and enhancement patterns of the solid components in our and previous cases included hypo- to isointensity on T1-weighted images, heterogeneous iso- to hyperintensity on T2-weighted images, and heterogeneous enhancement on contrast-enhanced imaging [4–7].

According to the imaging assessment in this study, all EMCs had well-demarcated margins and capsules, and a multinodular structure with internal septa was observed in four out of seven (57%) EMCs. Histologically, EMCs generally had well-circumscribed expansile borders and might be encapsulated by a well-defined fibrous capsule [9]. Our histological investigation revealed fibrous capsules in six lesions (86%), whereas the remaining one (14%) was surrounded by fibrous tissues. However, T2-weighted images demonstrated hypointense rims in all lesions. In other words, MRI could not distinguish tumor capsules from reactive fibrotic changes. Meanwhile, our histological investigation revealed well-demarcated margins in five lesions (71%), whereas the remaining two (29%) had ill-demarcated margins, because tumor cells microscopically extended beyond the capsule or fibrous tissue. However, MRI demonstrated well-demarcated margins in all lesions. It was assumed that the extension of the tumor cells beyond the capsule could not be captured by MRI.

Most EMCs histologically showed a characteristic multinodular growth pattern [2, 10, 11], and the glandular proliferation of EMCs were separated by dense fibrous connective tissues [10]. A histological multinodular growth pattern was also reported in other rare parotid gland lesions, such as oncocytoma [12] and sclerosing polycystic adenosis [13], but fibrous septa were not observed within the lesions. In addition, an MRI finding of multinodular growth pattern with internal septa has never been reported in frequently encountered parotid gland tumors [14–17]; therefore, it would contribute to the diagnosis of EMC. However, in terms of multinodular morphology and internal septa, histologically positive and MRI-negative cases were observed in two EMCs, respectively. In these cases, subtle histological findings could not be detected by MRI; therefore, it seemed to be a limitation of its spatial resolution.

In this study, the solid components on T2-weighted images were usually heterogeneous and demonstrated iso- to hyperintensity relative to the spinal cord. Our histological evaluation revealed various amounts of myxoid or chondroid matrix in all lesions; therefore, hyperintensity on T2-weighted images would reflect myxoid or chondroid matrices. In case 7, the reviewers determined the presence of cystic components due to marked hyperintensity on T2-weighted images and central unenhanced areas. However, our histological assessment revealed abundant chondroid matrix without cystic degeneration and necrosis.

Based on previous reports, the ADC values of EMCs were 0.92 [18] and 1.31 [5] × 10^−3^ mm^2^/s, and the mean or median ADC values of malignant parotid gland tumors were from 1.02 to 1.05 × 10^−3^ mm^2^/s [19–21]. In addition, the optimal ADC cutoff values of 1.315 [20] or 1.40 [21] × 10^−3^ mm^2^/s to distinguish parotid gland tumors between pleomorphic adenoma and malignancy were proposed. In contrast, in this study, the mean ADC value of EMCs was 0.967 × 10^−3^ mm^2^/s (range, 0.789–1.137 × 10^−3^ mm^2^/s); therefore, the ADC values of EMCs appeared to be typical ADC values for malignant parotid gland tumors.

The differential diagnosis of EMCs includes various subtypes of parotid gland tumors. Pleomorphic adenoma also appears as a well-defined hyperintense lesion on T2-weighted images due to their abundant myxoid or chondroid matrix [14]. Lobulated contours and fibrous capsules are common MRI features of pleomorphic adenomas and EMCs [14]. The mean maximum diameter of parotid pleomorphic adenomas (28.7 mm) [22] is substantially similar to that of EMCs in this study (29.1 mm). However, compared to pleomorphic adenomas, EMCs tend to have lower ADC values due to their higher cellularity and smaller amount of stromal matrix. In this study, the cystic degeneration also occurs more frequently in EMCs (43%) than in parotid pleomorphic adenomas (29%) [22]. Therefore, homogeneous hyperintensity on T2-weighted images is more common in pleomorphic adenomas (78%) [23] than in EMCs (14%). Although low-grade salivary gland cancers, including low-grade mucoepidermoid carcinoma and acinic cell carcinoma, usually exhibit hyperintensity on T2-weighted images, low-grade mucoepidermoid carcinomas frequently have ill-defined margins, reflecting peritumoral inflammatory changes [16]. Moreover, a multinodular structure with internal septa has not been reported in any other subtypes of parotid gland tumors.

According to a previous large clinical study that investigated the prognosis of 178 patients with parotid gland EMCs, the 5-year and 10-year overall survival rates were 89.5% and 88.1%, respectively [1]. Although local recurrence is not uncommon for EMCs, a long-term prognosis can be expected if patients receive appropriate treatment.

Previous case reports of EMCs described a preoperative diagnosis of pleomorphic adenoma based on fine needle aspiration cytology [9, 24]; therefore, its characteristic MRI features may play a complementary role for the diagnosis of EMC. Even if EMC is strongly suspected on MRI, a systemic screening would still be necessary because distant metastasis was observed in 25% of salivary gland EMCs. However, if MRI can accurately diagnose EMCs and deny high-grade salivary gland cancers, patients with EMC may avoid total or subtotal parotidectomy, elective nodal coverage, and elective neck irradiation, which are recommended for high-grade salivary gland malignancies [25]. Therefore, an accurate preoperative diagnosis of EMCs using MRI would lead to the selection of optimal treatment strategies and may avoid overtreatment.

This study has several limitations. First, the cohort was small because the study was conducted in a single institution. Second, this was a retrospective study, and all patients did not receive contrast-enhanced MRI. However, the characteristic MRI features were revealed in this study.

In conclusion, parotid gland EMCs usually appeared as predominantly solid lesions. Well-demarcated margins with complete or incomplete capsules were always demonstrated, and a characteristic multinodular structure with internal septa was frequently observed. The solid components showed heterogeneous iso- to hyperintensity relative to the spinal cord on T2-weighted images. The ADC values were consistent with those of other malignant parotid gland tumors. Although the MRI features of EMC could resemble those of benign salivary gland tumors or low-grade malignant salivary cancers, a multinodular structure with internal septa would be characteristic of EMC that may be useful in differentiating it from other parotid gland tumors.
